# Osteoporosis and Fragility Fractures in Patients with Liver Cirrhosis: Usefulness of FRAX^®^ as a Screening Tool

**DOI:** 10.3390/jcm13010188

**Published:** 2023-12-29

**Authors:** Jordi Sánchez-Delgado, Joaquim Profitós, Marta Arévalo, Alba Lira, Carlos Mármol, Mireia Miquel, Meritxell Casas, Mercedes Vergara, Xavier Calvet, Eugenio Berlanga, Luís del Rio, Oliver Valero, Ester Costa, Marta Larrosa, Enrique Casado Burgos

**Affiliations:** 1 Hepatology Unit, Digestive Disease Department, University Hospital Parc Taulí, I3PT Institute Research, Universitat Autònoma de Barcelona, 08208 Sabadell, Spain; jsanchezd@tauli.cat (J.S.-D.); alira@tauli.cat (A.L.); mmiquel@tauli.cat (M.M.); mcasasr@tauli.cat (M.C.); mvergara@tauli.cat (M.V.); xcalvet@tauli.cat (X.C.); 2 Centro de Investigación Biomédica en Red de Enfermedades Hepáticas y Digestivas, Instituto Carlos III, 28029 Madrid, Spain; 3 Hospital Consorci de Terrassa, 08227 Terrassa, Spain; jprofitos@cst.cat; 4 Rheumatology Department, University Hospital Parc Taulí, I3PT Institute Research, Universitat Autònoma de Barcelona, 08208 Sabadell, Spain; marevalo@tauli.cat (M.A.); ecostai@tauli.cat (E.C.); mlarrosa@tauli.cat (M.L.); 5 Hospital Sant Joan de Déu, 08950 Manresa, Spain; cjmarmol@althaia.cat; 6 Department de Medicina, Universitat de Vic—Universitat Central de Catalunya (UVic-UCC), 08500 Vic, Spain; 7 Clinical Analytics Department, University Hospital Parc Taulí, 08208 Sabadell, Spain; eberlanga@tauli.cat; 8 CETIR Ascires Centre Mèdic, 08029 Barcelona, Spain; ldelrio@cetir.es; 9 Department of Statistics, Universitat Autònoma de Barcelona, 08193 Bellaterra, Spain; oliver.valero@uab.cat

**Keywords:** liver cirrhosis, osteoporosis, bone mineral density, vertebral fracture, FRAX^®^, trabecular bone score

## Abstract

Purpose: The purpose of this study is to assess the prevalence of osteoporosis and fragility fractures in patients with liver cirrhosis (LC) and determine the associated risk factors, evaluating the usefulness of FRAX^®^ as a screening method to identify patients at a higher risk of fracture. Methods: This was a cross-sectional study. Demographic, clinical, and analytical data were collected in a randomized sample of LC patients attending the Hepatology Department of a university hospital. We assessed the absolute risk of fracture at 10 years (FRAX^®^) and based on the bone mineral density (BMD), the presence of morphometric vertebral fracture with a vertebral fracture assessment (VFA), or a thoracic and lumbar X-ray and bone microarchitecture with a trabecular bone score (TBS). Results: Ninety-two patients were included (71% male); the mean age was 63 ± 11.3 years. The main etiology of LC was alcoholism (52.2%), and most patients were Child–Pugh A (80.4%), with a mean model for end-stage liver disease (MELD) score of 10.1 ± 3.6. Sixteen patients (17.4%) had osteoporosis, and fifty-four (58.7%) had osteopenia. Eight patients (8.7%) had suffered at least one fragility fracture. The absolute risk of a major fracture according to FRAX without the BMD was 5.7 ± 4.5%. Risk factors associated with osteoporosis were age and the female sex. BMI > 30 was a protective factor. A FRAX cut-off point for a major fracture > 6.6% had a sensitivity of 69% and a specificity of 85% for a diagnosis of osteoporosis. Conclusions: The prevalence of osteoporosis and fractures in patients with LC is high, particularly in older women. FRAX^®^ may be a useful method to identify candidates for bone densitometry. A FRAX value below 6.6% without the BMD can avoid unnecessary testing.

## 1. Introduction

Hepatic osteodystrophy is the generic term defining the group of metabolic bone diseases, including osteoporosis and osteomalacia, which appear as possible complications of chronic liver disease [1].

Osteoporosis is very frequent worldwide and is the main cause of fragility fractures [2]. Osteoporosis-related fractures (mainly vertebral and hip) impose a substantial burden in terms of disability, costs, and mortality on postmenopausal women and older men. Patients with liver cirrhosis (LC), regardless of its etiology, have a higher prevalence of osteoporosis than the general population [3], and this condition has a major impact on morbidity, quality of life, and mortality. The prevalence of osteoporosis in LC varies widely, from 12% to 68% [1,4,5], and the fracture rate ranges from 5% to 20% [6]. This variation is predominantly due to differences in patient age, gender, and the etiology and severity of the liver disease [7,8,9]. The main risk factors for the development of osteoporosis in LC are vitamin D deficiency [10,11,12], hypogonadism [13], active alcohol consumption [10], chronic steroid treatment [14], low levels of insulin-like growth factor I [6,9], and a low body mass index (BMI) [11].

As the presence of osteoporosis in patients with LC is not routinely assessed, its prevalence is probably underestimated and therefore undertreated. Previous studies of the issue included few patients, mainly with advanced liver disease [15,16,17]. In a nationwide population-based study in China of 117,129 patients with hip fractures, including 4048 patients with LC, cirrhotic patients were relatively younger, their annual incidence of a hip fracture was seven times higher than the general population, they had higher complication rates due to infections and peptic ulcer disease, and their mortality rates at 1-year post-fracture were two to three times higher [18]. These results suggest that osteoporosis must be evaluated and detected early in patients with LC in order to minimize the risk of fractures and to improve their quality of life.

Several tools have been developed to help physicians in the assessment of a fracture risk, although the most frequently used worldwide is FRAX^®^ [19]. FRAX^®^ incorporates clinical variables (age, gender, BMI, history of previous fractures, family history of fracture, smoking, alcohol intake, rheumatoid arthritis, and glucocorticoid treatment) and the femoral neck BMD as an optional value [20]. FRAX^®^ estimates the 10-year absolute risk of a hip fracture and major osteoporotic fracture (hip, vertebral, humerus, and forearm). A country-specific FRAX threshold has been used in the general population to identify patients at a higher risk of fracture or to measure the BMD. When using FRAX for the Spanish population, the recommendation is to classify patients as being at a high risk of fracture when the FRAX for a hip fracture is ≥3% or when the FRAX for a major fracture is ≥10% without the BMD or ≥7.5% with the BMD [21]. In addition, a FRAX for a major fracture > 5% could be useful to select patients who are suitable for DXA [19]. However, there is no well-defined threshold for testing the BMD or for classifying patients with LC as being at a high risk of fracture. Although guidelines of some gastroenterology and hepatology societies recommend a DXA scan in all cirrhotic patients [22,23], this strategy could be expensive and unproductive in many patients. As FRAX is simple to use, reproducible, and can be repeated at each patient visit, it could be a useful tool to identify LC patients with a higher risk of osteoporosis and fractures, avoiding unnecessary DXA scans.

The aim of our study was to assess the prevalence of osteoporosis and fragility fractures in patients with LC, to analyze the main risk factors, and to determine the usefulness of FRAX^®^ as a screening method to identify patients at a higher risk of fractures.

## 2. Materials and Methods

### 2.1. Study Patients

This was a single-center, cross-sectional study of patients with LC identified through ICD-9-CM Diagnosis Code 571.5 among all patients attending the Hepatology Department of the university hospital Parc Taulí in Sabadell (Barcelona). To obtain an unbiased representation of the total population, a random sampling was performed first, in which each sample had an equal probability of being chosen. From this random sample, we selected all patients who met the following inclusion criteria: age over 18 years old; diagnosis of cirrhosis via liver biopsy (METAVIR stage F4) and/or transient elastography (Fibroscan^®^, Echosens, France, FibroScan Probe XL+ Class: IIa.); published cut-off values for different etiologies and/or ultrasound evidence of a nodular liver and/or portal hypertension and/or based on specific clinical manifestations of decompensation (ascites, hepatic encephalopathy, or variceal bleeding); assessment of severity of the underlying liver disease using the Child–Pugh and the model for end-stage liver disease (MELD) scores; provision of signed informed consent; ability to understand and complete the questionnaires administered.

The exclusion criteria were as follows: pregnancy; disorders that might influence the accuracy of DXA, overweight > 120 kg; inability to perform DXA; end-stage renal disease, autoimmune diseases, such as rheumatoid arthritis, ankylosing spondylitis, inherited connective tissue disorders, sarcoidosis, amyloidosis, and HIV infection. Finally, we also excluded patients with diagnosis of LC secondary to primary biliary cholangitis because its prevalence among patients with cirrhosis is low, and the mechanisms and factors involved in the pathogenesis of osteoporosis are already widely known.

Patients receiving osteoporosis treatment (including calcium, vitamin D, antiresorptives, and osteoanabolics) were not excluded in order to calculate the real prevalence of osteoporosis and fractures in the whole population of LC patients. Patients with and without vitamin D supplementation were analyzed separately in order to avoid a bias in the interpretation of vitamin D deficiency as a potential risk factor.

### 2.2. Variables

In all selected patients, demographic, clinical, and analytical data were collected; a DXA scan was performed; and a FRAX score for Spanish population was calculated (https://www.sheffield.ac.uk/FRAX/tool.aspx?lang=sp, accessed on 22 November 2021).

Demographic and clinical data included the following: birth date, sex, height and weight for body mass index (BMI) measurement, etiology of cirrhosis, smoking status, alcohol intake (unsafe alcohol consumption was defined as >14 units of alcohol/week (1 unit of alcohol = 10 mL (8 g) of pure alcohol) for women and >21 units of alcohol/week for men, post-menopausal status for women (amenorrhea > 12 months), history of fragility fractures, and clinical parameters to calculate the Child–Pugh and MELD scores. The Child–Pugh score considers levels of bilirubin, INR, and albumin and the presence of ascites or hepatic encephalopathy. The MELD score includes INR, creatinine, and also bilirubin. Any data missing from the clinical record were obtained from the patient survey questionnaire.

Laboratory analysis was performed at the same time as the DXA scan and comprised liver and renal function tests; biochemical parameters, including serum levels of calcium, phosphorus, parathyroid hormone (PTH), and total and free testosterone; 25 hydroxyvitamin D; 24-h urine calcium; serum cross-linked C-telopeptide of type I collagen (CTX) (marker of bone resorption) (Elecsys, Roche Diagnostics, Mannheim, Germany); and bone-specific alkaline phosphatase (BAP) (marker of bone formation) (Beckman Coulter Acces Ostase Assay).

Serum levels of 25-hydroxyvitamin D were measured using LC-MS/MS (TSQ Quantum Access MAX, Thermo Fisher Scientific, Waltham, MA, USA), and an analytical sensitivity of 4 ng/mL 25-hydroxyvitamin D > 30 ng/mL was considered normal, 20–30 ng/mL was considered insufficiency, and <20 ng/mL was considered deficiency.

CTX was measured using electrochemiluminescence automated immunoassays (Elecsys, Roche Diagnostics, Mannheim, Germany), with an analytical sensitivity of 70 ng/L; BAP was measured with ELISA (Immunodiagnostic Systems, Boldon, UK), with an analytical sensitivity of 0.7 μg/L.

We investigated the presence of prevalent vertebral fractures through VFA. In cases with difficult interpretation, we performed a spine X-ray.

FRAX for major fracture and for hip fracture was calculated in all patients with and without the femoral neck BMD. We considered three or more units/day of alcohol consumption in all patients with alcoholic cirrhosis, regardless of current alcohol intake. For the calculation of FRAX, we followed the manufacturer instructions and considered patients affected by secondary osteoporosis since they suffered from a disorder strongly associated with osteoporosis (chronic liver disease).

Subjects with BMI below 18.5 kg/m^2^ were classified as underweight, those with BMI 18.5 to 24.9 kg/m^2^ as normal or healthy weight, those with BMI 25.0 to 29.9 kg/m^2^ as overweight, and those with BMI above 30.0 kg/m^2^ as obese.

BMD at lumbar spine (L1–L4), femoral neck, and total hip was measured with DXA (Prodigy Series X-ray; GE Medical systems Lunar, Madison, WI, USA) and expressed in grams per square centimeter. In addition, all values were expressed as T score, as absolute numbers defined in 1994 by WHO (amount of standard deviations below or above the mean bone density value of young white Caucasian women at peak bone mass). The precision of the DXA measurement and the coefficient of variation (CV) at the level of the lumbar spine was 1% and at the hip was 1.5%. We used the WHO criteria [24] to define osteopenia (T score between −1 and −2.5) and osteoporosis (T ≤ −2.5) at any of the three sites. For T-score values we used the specific software Spanish population database included in DXA equipment.

DXA was performed after paracentesis in patients with decompensated cirrhosis and suspected ascites fluid volume of more than 4 L [25]. Clinical osteoporosis was defined if there were any previous vertebral or non-vertebral fragility fractures (non-traumatic fracture or fracture after a low energy trauma such as a fall from standing height), regardless of BMD.

Trabecular bone score (TBS) via TBS insight^®^ (Medimaps SA, Canéjan, France) was also analyzed in all patients. TBS is an indirect measurement of trabecular bone microarchitecture that can be determined from DXA images using a specific analysis software, and it is an independent fracture risk [26]. TBS > 1.31 was considered normal, between 1.31 and 1.23 reflected partial deterioration of bone microarchitecture, and <1.23 degraded microarchitecture.

Demographic, clinical, and laboratory data; DXA performance; VFA; spine X-ray; TBS; and FRAX calculations were performed over a 2-week period.

### 2.3. Statistical Analysis

Demographic data, including age, sex, weight, height, and BMD are presented as mean ± SD or median with range. Potential risk factors for osteoporosis were analyzed by subgroups (according to age, sex, BMI, and LC etiology). Biochemical markers of bone metabolism were assessed, and the differences in the demographic characteristics of the two groups were examined using the *t* test. Multiple logistic regressions were used to determine the associations between osteoporosis and age, sex, etiology of the cirrhosis, BMI, biochemical findings, and FRAX score. Data management and statistical analyses were performed using SAS v9.4, with a *p* < 0.05 indicating statistical significance.

### 2.4. Ethical Considerations

All patients provided signed written informed consent. The protocol was approved by the Ethics Committee of Parc Taulí University Hospital (CEIM HPT: 2015540). ClinicalTrials.gov Identifier: NCT03201016.

Patients’ data were stored in a database in which their identity was protected and which only the principal investigator was able to access.

## 3. Results

### 3.1. Baseline Characteristics

Seven hundred and sixty patients diagnosed with LC were identified as “cirrhosis” in our database (CIE 9 571). The computer selected 150 patients randomly. Seventeen patients did not meet the inclusion criteria or exclusion criteria, three were clinic “non-attenders”, five had the incapacity to perform DXA, six patients did not want to participate, and nine patients were excluded for other reasons. Finally, one hundred and ten patients were considered eligible and signed the informed consent. Eighteen patients did not attend the scan. Ninety-two patients were finally included. Patients selected by the computer were visited from November 2015 to September 2017.

Demographic and clinical characteristics are shown in Table 1. The median age was 63 ± 11.3 years, and 70.1% were men. Alcohol was the most common cause of cirrhosis (52.2%), followed by HCV infection (27.2%), mixed HCV infection, and alcohol (8.7%). In all, 80.4% of patients were Child A. Median (range), the MELD score was 10.1 (SD: 3.6), and the median BMI was 29.6 kg/m^2^. Only seven patients (7.6%) had received >5 mg of prednisone or its equivalent for three or more months before enrollment. Only one patient required paracentesis before performing DXA due to refractory ascites. The baseline analytical data of cirrhotic patients are shown in Table 2 and the BMD and TBS values in Table 3.

### 3.2. Prevalence of Osteoporosis and Fragility Fractures

Sixteen patients (17.4%) had osteoporosis, fifty-four (58.7%) had osteopenia, and twenty-two (23.9%) had a normal BMD. Eight patients (8.7%) had suffered previous fragility fractures (mainly vertebral). Nineteen patients (20.7%) had clinical or densitometric osteoporosis (osteoporosis defined by DXA and/or fragility fracture) Table 4.

### 3.3. Prevalence of Vitamin D Deficiency

The mean levels of 25-hydroxyvitamin D in the global cohort were 18.48 ng/mL ± 9.79 (16.31 ng/mL ± 8.06 among patients without supplementation [*n* = 77]). An amount of 86.0% of patients had low vitamin D levels (<30 ng/mL); 21.6% had vitamin D insufficiency (20–30 ng/mL); 64.0% had vitamin D deficiency (<20 ng/mL); and 31.1% had severe deficiency (≤10 ng/mL).

### 3.4. Prevalence of Trabecular Bone Microarchitecture Deterioration according to TBS

The median TBS was 1.18 (range 0.89–1.70). Fifty-seven patients (62%) had a degraded bone microarchitecture (TBS < 1.23). Ten patients (11%) had a partial deterioration of bone microarchitecture, and twenty-five (27%) had a normal TBS.

### 3.5. Clinical Factors Associated with Osteoporosis and Fracture

A bivariate analysis between patients with or without osteoporosis via DXA showed that age (*p* < 0.001) (OR 1.14) and the female sex (*p* = 0.046) (OR 4.5) were the only independent risk factors for osteoporosis in our cohort of cirrhotic patients (Table 5). On the other hand, a BMI > 30 was found to be a protective factor (*p* = 0.012).

None of the analytical parameters, including calcium, phosphorus, 25-hydroxyvitamin D, PTH, CTX, and BAP, were associated with a higher risk of osteoporosis.

Fractures were statistically more frequent in cirrhotic patients with osteoporosis (*n* = 5, 31.3%) than in patients with osteopenia or normal DXA (*n* = 3, 3.9%), *p* < 0.001. A BMD in the range of osteoporosis was the only factor associated with fracture.

### 3.6. Usefulness of FRAX in the Prediction of Osteoporosis and Fracture

The absolute risk of a major fracture (vertebral, proximal humerus, hip, or distal radius) at 10 years according to a FRAX without a femoral neck BMD in the whole cohort was 5.76 ± 5.77%, and with a BMD, it was 4.9 ± 5%. The risk of a hip fracture without a femoral neck BMD was 2.53 ± 4.25%, and with a BMD, it was 2.07 ± 3.65%. The mean 10-year risk of a major fracture and hip fracture among patients with osteoporosis was significantly higher than in patients with osteopenia or a normal BMD. After adjusting FRAX with TBS, the mean risk of a hip and major fracture increased in both groups (Table 6).

**Figure 1:**
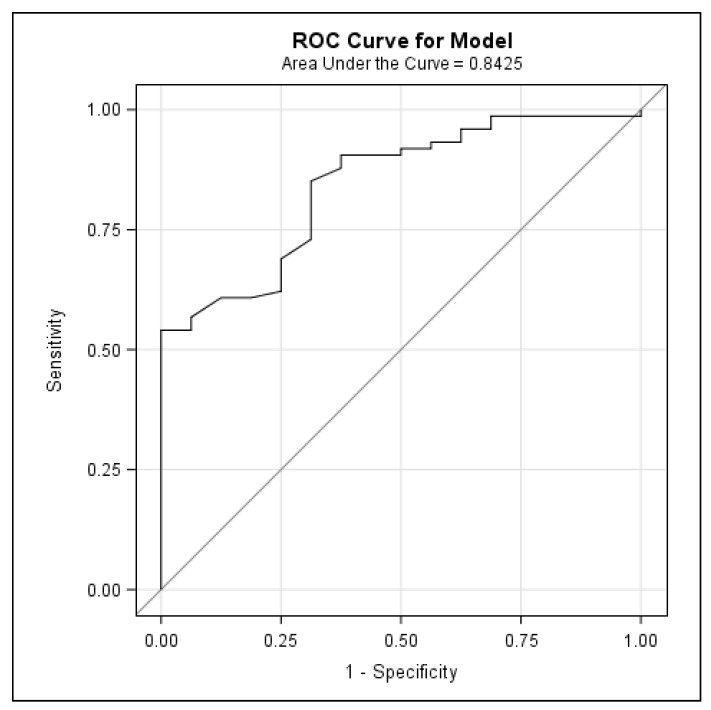
ROC curve model for major fracture for FRAX without BMD and diagnosis of osteoporosis.

An ROC curve model was used to identify the best threshold of a FRAX major fracture to identify patients with osteoporosis. A cut-off for a FRAX major fracture without a BMD of >6.6% had a sensitivity of 69% and a specificity of 85% for the diagnosis of osteoporosis, with a positive predictive value of 50% and a negative predictive value of 93% (Figure 1).

## 4. Discussion

We found that patients with LC, even in the early stages, had a high prevalence of a low BMD, with 17.4% of patients having osteoporosis via DXA. Nearly 9% of patients had some previous fragility fractures, mainly vertebral fractures. These results are striking given that most of the included patients had preserved liver function (80.4% were on the Child A stage).

This high prevalence of osteoporosis in our cohort may also be related to the high rate of cirrhosis due to alcohol (*n* = 56, 61%), which is a known risk factor for osteoporosis [10,27]. The prevalence of osteoporosis in patients with LC is expected to rise in the coming years due to the increased prevalence of cirrhosis due to alcohol consumption after the elimination of HCV infection and control of chronic hepatitis B. A recent study reported a prevalence of osteoporosis in cirrhotic patients (mixed etiology) ranging from 12% to 39% and the presence of fractures in between 7% and 35% [28]. In a study of patients on the liver transplant list, Nincovik et al. reported osteoporosis in 36.6%, osteopenia in 48.1%, and a normal BMD in only 15.2% [29]. These rates are higher than ours, probably due to selection bias (their patients were on the transplant waiting list and probably had more advanced hepatic insufficiency). Overall, the results support the idea of establishing the detection of osteoporosis in all patients with LC as a routine measure.

Another interesting finding in our study was the fact that the severity of liver disease, assessed with Child–Pugh class or MELD score, was not associated with osteoporosis. The mean BMD and T scores in Child A patients did not differ significantly from those of their Child B or C peers. Eighty percent of our patients were Child A, a rate that reflects the real picture of our clinical practice and allows us to establish detection and prevention protocols. However, the results obtained in other studies are controversial. Osteoporosis, assessed with the DXA criteria, was found in 24−38% of patients with end-stage disease, depending on the series [15,30]. In addition, patients with end-stage liver disease frequently suffer fractures; indeed, 6.6−36% of candidates for liver transplantation had radiological vertebral fractures [31,32]. In contrast, other studies did not find a correlation between the BMD and the clinical severity of cirrhosis [29,33]. Nevertheless, the message seems to be that we should not wait until the advanced stages of liver disease before assessing bone health in LC patients.

We found that the female sex and age were significant risk factors for osteoporosis, while a BMI > 30 kg/m^2^ was a protective factor. These data are consistent with previous reports in the literature, both in the general population and in patients with chronic liver disease [34,35]. Even though most studies suggest that obesity has a favorable effect on BMD, its effect on skeletal microarchitecture is unclear. In previous studies, factors associated with osteoporosis were gender, advanced age, previous fragility fracture, menopause, male hypogonadism, immobilization or physical inactivity, excess alcohol intake, low body mass index, chronic cholestasis, end-stage liver disease, long-term corticosteroid therapy (>5 mg for more than three months), and immunosuppressive agents [36]. Although we analyzed all these variables, we did not find any additional associations, probably due to the low number of patients with these characteristics.

25-Hydroxivitamin D deficiency is highly prevalent in patients with chronic liver disease [37,38]. In our study, more than 90% of patients had low levels of 25-hydroxyvitamin D, and 31.1% had a severe deficiency. Nevertheless, these low values were not associated with the presence of osteoporosis, so the prevalence of vitamin D deficiency is universal in patients with LC, regardless of their BMD. A recent study revealed that 90% of patients with alcoholic liver cirrhosis have vitamin D inadequacy (<80 nmol/L), with the lowest serum 25-hydroxyvitamin D levels being recorded in patients with Child–Pugh stage C [39]. One study reported that, among the patients with alcoholic cirrhosis, 85% had serum vitamin D levels below 50 nmol/L and 55% had levels below 25 nmol/L [40].

As far as we are concerned, this is the first study analyzing TBS in a large cohort of cirrhotic patients. It is remarkable that, in our study, a high proportion of our cohort had a degraded bone microarchitecture, as reflected by TBS values (Table 3). It means that, probably, LC may harm bone quality in higher proportion than bone density. Wakolbinger R. et al. analyzed TBS in small cohort of 19 patients with LC, finding a TBS value decrease of 17% in the patients compared with the controls [41].

In our study, 8.7% of the patients presented previous fragility fractures, a rate similar to that reported elsewhere. However, a spine X-ray was not performed in all the patients to detect a morphometric vertebral fracture, and so, the real prevalence of vertebral fractures may be higher. In a recent systematic review and meta-analysis, the pooled ORs of the association between LC and any fracture risk, hip fracture, spine/trunk fracture, and limb fracture were 1.94 (95% CI, 1.59–2.37), 2.11 (95% CI, 1.34–3.32), 2.00 (95% CI, 1.50–2.67), and 1.82 (95% CI, 1.65–2.01), respectively [42]. In a population-based study with more than 20,000 patients with alcoholic cirrhosis in England and Denmark, rates of hip fractures were five times higher (adjusted HR 5.5; 95% CI 4.3–6.9) than average in the English patients and 8.5 times higher (adjusted HR 8.5; 95% CI 7.8–9.3) in the Danish patients [43].

Finally, this is the first study to establish a FRAX threshold to identify cirrhotic patients who are at a high risk of osteoporosis and are therefore candidates for BMD testing. An absolute 10-year risk for a major fracture with a FRAX > 6.6% identifies patients with LC who will benefit from bone densitometry. While a BMD using DXA is an important predictor of fracture risk, its predictive value is constrained by the lack of information on clinical risk factors for fractures. Composite scores, including the BMD and validated clinical criteria, such as the FRAX tool, can help to estimate the future risk of fractures and to establish the trigger value for performing DXA. The FRAX tool should be routinely used, as it is easy to apply in the outpatient and inpatient settings. In our study, the mean FRAX scores were 5.76% ± 5.77 for major osteoporotic fracture and 4.95% ± 4.96 when including the femoral neck BMD. To our knowledge, only two other studies have used the FRAX tool to predict the risk of fracture in cirrhotic patients [44,45]. In the study by Casanova-Lara of 52 cirrhotic patients, including cholestatic etiology, the 10-year fracture risk with FRAX was 7.77% ± 6.71, rising to 13.72% ± 1 when the value of the femoral neck BMD was added. De et al. found a similar result using the FRAX tool to rule out osteoporosis in Indian cirrhotic patients, recording 10-year probabilities of a major osteoporotic fracture and hip fracture of 5.7% (2.1–28.9) and 2.5% (1.4–7.4), respectively. Using FRAX probability cut-offs of 20% for a major osteoporotic fracture and 3% for a hip fracture, 30% of patients qualified for osteoporosis treatment [45]. In our study, with a 6.6% cut-off for FRAX, we could have avoided performing 75% of all DXA scans (68 of 92 explorations), which would have represented a major financial saving and would also have saved the patient considerable discomfort. Half of our patients with a FRAX for a major fracture > 6.6% had osteoporosis via DXA and were therefore eligible for bone-specific treatment.

Our study has several strengths. First, it is a study with a random sample of patients diagnosed with LC, including a large proportion of patients with preserved liver function; hence, the prevalence of osteoporosis and fractures is in the overall population of LC patients. Another strength is that also morphometric vertebral fractures were assessed. It is also the first study to analyze the usefulness of the FRAX tool to select DXA candidates and avoid unnecessary tests to assess the BMD. Finally, it is also the first study giving TBS data in a large cohort of LC patients.

### 4.1. Limitations of the Study

Our study has also some limitations. We did not have a control group. We did not exclude patients with other clinical factors associated with osteoporosis or fractures, such as diabetes, early menopause, or steroid use, among others, nor did we exclude patients receiving osteoporosis treatment. It was in order to calculate the real prevalence of osteoporosis and fractures in the global population of LC patients. In fact, although FRAX has better accuracy for untreated patients, some studies reported that it can also be used to predict the fracture probability in patients currently or previously treated for osteoporosis [46]. For the same reason of having data of the global adult population of LC, we did not exclude two patients under 40 years old (31 and 34 years) despite the fact that FRAX can only be calculated in patients over 40 years of age. A FRAX calculation in those patients was assuming 40 years of age, resulting equally in a very low fracture risk (<2% of a major fracture and 0% of a hip fracture in both patients). Finally, we know that BMD and TBS measurements could be affected by abdominal thickness. Although the DXA scan was performed after paracentesis in patients with decompensated cirrhosis and suspected ascites fluid volume of more than 4 L, we cannot exclude a bias in BMD and TBS measurements in patients with mild-to-moderate ascites.

### 4.2. Conclusions

The prevalence of osteoporosis in patients with LC is high, even in those with a preserved liver function (Child A and/or a low MELD score). Age and the female sex are factors significantly associated with the presence of osteoporosis, while a BMI > 30 could be protective. Most of the LC patients have a degraded bone microarchitecture according to TBS. Although assessing the BMD and TBS seems to be useful in patients with LC, it seems more efficient to select patients with a 10-year risk of a major fracture over 6.6% with FRAX for screening for osteoporosis via DXA.

## Figures and Tables

**Table 1:** Baseline demographic and clinical characteristics of study cohort.

Baseline Characteristics	*n* = 92
Age, mean, years, (SD) (range)	63 (11.3) (31–85)
Male/female, *n* (%)	65 (71%)/27 (29%)
Alcohol consumption, *n* (%)	
Active	15 (16.3%)
Former	42 (45.7%)
Never	35 (38%)
Cause of cirrhosis, *n* (%)	
Alcohol	48 (52%)
HCV	25 (27.2%)
Alcohol + HCV	8 (8.7%)
HBV	4 (4.3%)
Autoimmune hepatitis	3 (3.3%)
MASLD	3 (3.3%)
Cardiac	1 (1.1%)
Child Pugh class, *n* (%)	
A	74 (80.4%)
B	16 (17.4%)
C	2 (2.2%)
Previous decompensation (yes)	36 (39.1%)
MELD score (SD)	10.14 (3.6)
BMI, Mean	
Normal weight	18 (19.6%)
Overweight	38 (41.3%)
Obesity	36 (39.1%)
Osteoporosis main risk factors (%)	
Current smoker	26 (28.3%)
Alcohol consumption ≥ 3 U/d	57 (62.0%)
Current or previous exposure to corticoids	7 (7.6%)
Rheumatoid arthritis	1 (1.1%)
Family history of hip fracture	11 (12.6%)
Early menopause	8/27 (29.63%)
Calcium supplementation	15/84 (17.8%)
Vitamin D supplementation	15/87 (17.2%)
Oral bisphosphonates	7 (7.6%)
Other osteoporosis treatment	
Zoledronic e.v	1 (1%)
Teriparatide	1 (1%)

HCV: Hepatitis C virus; HBV: Hepatitis B virus; MASLD: Metabolic (dysfunction) associated fatty liver disease; MELD: Model for End Stage Liver Disease; BMI: Body Mass Index.

**Table 2:** Baseline laboratory parameters of the study cohort.

Variable	*n*	Mean	SD	Normal Values
Leukocytes (×10^9^/L)	92	5833.59	2309.99	4000–11,000
Hemoglobin (g/L)	92	135.66	23.65	130–175
Platelets (×10^9^/L)	92	129,119.57	50,426.72	130–400,000
Urea (mg/dL)	92	35.27	20.80	10–50
Creatinine (mg/dL)	92	0.88	0.26	0.7–1.2
Aspartate transaminase (U/L)	92	36.18	35.09	0–38
Alanine transaminase (U/L)	92	29.83	43.36	10–41
Alkaline phosphatase (U/L)	92	97.23	52.15	40–129
Gamma glutamyl transpeptidase (U/L)	92	84.26	90.39	8–61
Total bilirubin (mg/dL)	92	1.18	0.89	0.1–1.3
INR (ratio)	92	1.28	0.43	0.7–1.2
Calcium (mg/dL)	84	9.44	0.50	8.8–10.2
Corrected calcium (mg/dL)	84	9.35	0.39	8.8–10.2
Phosphorus (mg/dL)	80	3.15	0.53	2.7–4.5
Magnesium (mg/dL)	85	1.96	0.21	1.6–2.6
Total proteins (g/L)	85	69.84	9.26	66–87
Albumin (g/L)	91	41.46	5.80	34–48
Parathormone (pg/mL)	88	51.86	23.09	10–65
25-hydroxyvitamin D (ng/mL)	87	18.48	8.06	20–40
24-h urine calcium (mg/24 h)	72	125.63	90.75	100–320
Total testosterone (nmol/L)	55	5.69	2.95	1.93–7.4
Vitamin K (ng/mL)	86	1.00	1.87	0.2–3.2
Iron (µg/dL)	84	93.85	44.61	60–158
Ferritin (ng/mL)	85	141.80	171.50	30–400
Transferrin saturation (%)	83	31.44	33.05	30–55
CTX (ng/mL)	84	0.33	0.19	0.016–0.584
BAP (U/L)	86	17.85	8.44	0–20

CTX: Serum cross-linked C-telopeptide of type I collagen. BAP: Bone alkaline phosphatase.

**Table 3:** Bone Mineral Density (BMD) and Trabecular Bone Score (TBS).

Variable	Median	Range
Lumbar spine BMD (g/cm^2^)	1.15	(0.23–0.59)
Lumbar spine T-score	−0.72	(−5.06–1.82)
Femoral neck BMD (g/cm^2^)	0.88	(0.14–0.52)
Femoral neck T-score	−1.25	(−3.82–1.07)
Total hip BMD (g/cm^2^)	0.95	(0.15–0.53)
Total hip T-score	−0.91	(−3.95–1.15)
Trabecular bone score (TBS)	1.18	(0.89–1.70)
TBS T-score	−3.20	(−6.5–2.02)

**Table 4:** Prevalence of osteoporosis and fragility fractures.

	*n*	%
Osteoporosis (T-score ≤ −2.5)	16	17.4
Osteopenia (T-score between −1 and −2.5)	54	58.7
Normal BMD (T-score ≥ −1)	22	23.9
Osteoporosis (clinical or densitometric)	19	20.7
Fragility fractures	8	8.7
Vertebral	6	6.5
Hip	2	2.2

**Table 5:** Clinical characteristics of cirrhotic patients with and without osteoporosis via DXA.

Clinical Characteristics	Osteoporosis (*n* = 16)	Normal BMD/Osteopenia (*n* = 76)	*p* Value
Age (years) (mean ± SD)	72.25 ± 9.09	61.14 ± 10.77	<0.001
Gender (female)	8 (50%)	19 (25%)	0.04
MELD score (mean ± SD)	11 ± 3.9	9.9 ± 3.56	0.35
CHILD-PUGH (A) (number of patients)	14 (87.5%)	60 (78.9%)	0.43
Active alcohol consumption (number of patients)	2 (12.5%)	13 (17.1%)	0.08
Current smoking (number of patients)	2 (12.5%)	24 (31.5%)	0.13
BMI (>30 kg/m^2^) (number of patients)	10 (62.5%)	28 (36.8%)	0.01
Diabetes mellitus (number of patients)	4 (21.1%)	15 (78.9%)	0.63
CKD (number of patients)	2 (12.5%)	6 (7.9%)	0.55
Hyperparathyroidism (number of patients)	1 (6.2%)	4 (5.2%)	0.54
Early menopause (number of patients)	2 (12.5%)	6 (7.9%)	1
Use of low molecular weight heparin (number of patients)	3 (18.7%)	5 (6.6%)	0.11
Use of glucocorticoids (number of patients)	1 (6.2%)	6 (7.9%)	0.82

**Table 6:** Association between FRAX (without and with femoral neck BMD and adjusted with TBS) with densitometric osteoporosis and normal BMD/osteopenia.

Clinical Characteristics	Osteoporosis (*n* = 16)	Normal BMD/Osteopenia (*n* = 76)	*p* Value
FRAX MOF (without BMD)	10.92 ± 8.29	4.65 ± 4.41	<0.001
FRAX MOF (with BMD)	9.84 ± 7.43	3.89 ± 3.51	<0.0001
FRAX HIP fracture (without BMD)	5.6 ± 5.02	1.87 ± 3.78	<0.0001
FRAX HIP fracture (with BMD)	5.08 ± 4.78	1.43 ± 3.02	<0.0001
FRAX MOF (adjusted with TBS)	12.37 ± 7.67	5.3 ± 3.89	<0.0001
FRAX HIP fracture (adjusted with TBS)	5.83 ± 4.72	1.92 ± 3.14	<0.0001

MOF: Major osteoporotic fracture.

## Data Availability

The datasets generated during and/or analyzed during the current study are available from the corresponding author on reasonable request.

## Abbreviations

LC	Liver cirrhosis
BMD	Bone mineral density
TBS	Trabecular Bone Score
DXA	Dual-energy X-ray Absorptiometry
BMI	Body Mass Index
MELD	Model for End-Stage Liver Disease
